# Asymmetric proptosis in thyroid eye disease

**DOI:** 10.1007/s10792-024-03141-6

**Published:** 2024-04-27

**Authors:** Khizar Rana, Devanshu Garg, Lee Shien S. Yong, James Leyden, Sandy Patel, James Slattery, Garry Davis, Weng Onn Chan, Dinesh Selva

**Affiliations:** 1 Department of Ophthalmology and Visual Sciences, University of Adelaide, North Terrace, Adelaide, SA 5000 Australia; 2 South Australian Institute of Ophthalmology, Royal Adelaide Hospital, Port Road, Adelaide, SA 5000 Australia; 3 Department of Medical Imaging, Royal Adelaide Hospital, Port Road, Adelaide, SA 5000 Australia

**Keywords:** Thyroid eye disease, Asymmetry, Orbit

## Abstract

**Purpose:**

Patients with thyroid eye disease (TED) can present with asymmetric disease. The purpose of this study was to evaluate the prevalence of asymmetric TED in an Australian cohort and investigate clinical, biochemical, and radiological associations of asymmetric TED.

**Methods:**

This was a retrospective study of patients with TED who underwent Hertel exophthalmometry and orbital computed tomography (CT) scans. Asymmetry was defined as a difference in the globe protrusion of ≥ 3 mm using Hertel exophthalmometry. Data was collected on patient demographics, clinical disease presentation, thyroid function tests and antibody levels. Muscles volumes were determined by manually segmenting the extraocular muscles on CT scans using a commercially available software.

**Results:**

172 orbits from 86 patients were included in the study. 28 (33%) patients had asymmetric TED. No significant differences were observed in age, gender, thyroid hormone status, anti-thyroid peroxidase levels, thyroid stimulating hormone receptor antibodies, disease activity status, or dysthyroid optic neuropathy between the asymmetric and non-asymmetric groups. The extraocular muscle volumes were significantly higher in the asymmetric orbit compared to its contralateral orbit.

**Conclusion:**

Asymmetric TED is a reasonably common occurrence in the course of TED. It is associated with higher extraocular muscle volumes, suggesting muscle enlargement as one of the underlying contributors to asymmetric proptosis. Thyroid eye disease should be considered in the differential of asymmetric orbital inflammatory disease.

## Introduction

Thyroid eye disease (TED) is an autoimmune condition characterised by inflammation of the orbital tissues, resulting in ocular manifestations such as proptosis, diplopia, eyelid retraction, and optic neuropathy. While the disease manifests bilaterally and symmetrically in most cases, asymmetric involvement of the orbits has been reported. Asymmetry has been identified as a significant contributor to psychosocial distress and quality of life [1]. Asymmetric TED may represent a distinct variant of TED and has been reported to be present in 9–41% of patients [1, 2]. Asymmetric presentation of TED may pose diagnostic challenges; thus it is important to be aware of its clinical, biochemical and radiological associations. Conflicting results have been reported with regards to the clinical and biochemical associations of asymmetric TED. Some studies have shown a correlation between asymmetry and male gender, older age, hypothyroid/euthyroid status, and severe disease, whilst others have failed to detect such differences [3]. Thus, the purpose of our study is to evaluate the prevalence of asymmetric TED in an Australian cohort, and determine its clinical, biochemical and radiological associations. This may help contribute to the broader understanding of asymmetric TED.

## Methods

We included patients who were diagnosed with thyroid eye disease (TED) and had Hertel exophthalmometry and orbital computed tomography (CT) scans. The diagnosis of TED was made by orbital surgeons or neuro-ophthalmologists, using established criteria [4]. Asymmetry was defined as a difference in the globe protrusion difference of greater than or equal to 3 mm using Hertel exophthalmometery [5]. Hertel exophthalmometry was performed by Orbital surgeons or Neuro-Ophthalmologists. Disease activity was classified as active or inactive, and the presence or absence of dysthyroid optic neuropathy was recorded. Thyroid status was categorised based on thyroid function tests as hyperthyroid, subclinical hyperthyroidism, euthyroid, or hypothyroid. The study was approved by local research ethics committee and adhered to the tenets of the Declaration of Helsinki.

Multi-detector CT scanners were used to obtain orbital CT scans 0.6 mm thick at 0.4 mm intervals following standard imaging protocols. The analysis of CT images was carried out using OsiriX software (version 11.0, Pixmeo SARL, Switzerland) for the purpose of calculating muscle volume. The extraocular muscles were manually segmented in consecutive axial and coronal slices to determine their volumes. To accomplish this, the brush tool in the software was used to outline the muscles on each slice, and the total volume was calculated by summing the areas in each slice and multiplying by the slice thickness. The sum of all muscles was obtained by adding all of the individual muscle volumes in an orbit.

Statistical analysis was performed using Stata software (version 13.0, StataCorp LLC, USA). Descriptive statistics were used to summarise patient demographics and clinical characteristics. The independent *t*-test was used to determine the difference in the two groups in terms of age, anti-thyroid peroxidase (anti-TPO) levels, thyroid stimulating hormone receptor antibodies (TSHrAb) and muscle volumes. Pearson’s Chi-squared test was used to compare categorical variables between the two groups. A *p* value less than 0.05 was considered statistically significant.

## Results

**Fig. 1:**
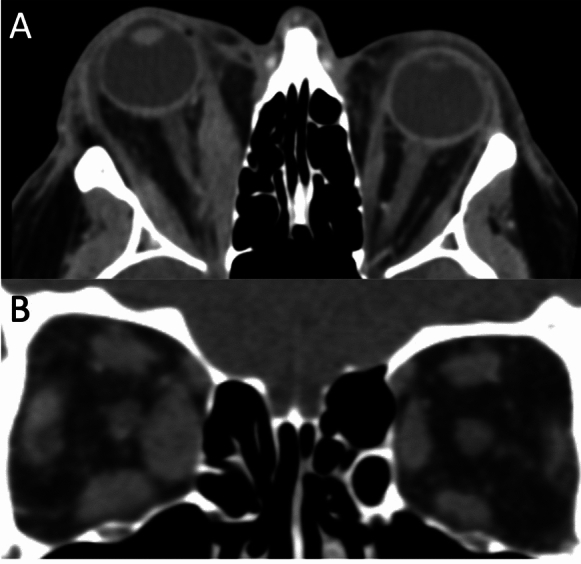
**A** Axial CT demonstrating right axial proptosis and medial rectus enlargement. **B** Coronal CT showing enlargement of the right extraocular muscles, with preserved left extraocular muscles

172 orbits from 86 patients were included in the study. Twenty-eight patients (33%) had asymmetric TED. The mean age was 54 ± 16 years, with 57 females and 29 males. No significant differences were found in mean age (asymmetric 54.7 vs. non-asymmetric 53.7 years old, *p* = 0.79) or gender (asymmetric 65% female vs. non-asymmetric 67% female, *p* = 0.07). Similarly, no significant differences were observed in thyroid hormone status (*p* = 0.16), anti-TPO levels (*p* = 0.38), TSHrAb levels (*p* = 0.14), disease activity status (active TED 11/28 in asymmetric group vs. 48/144 in symmetric group, *p* = 0.37), or dysthyroid optic neuropathy (5/28 in asymmetric group vs. 15/144 in symmetric group, *p* = 0.26) between the two groups. The extraocular muscle volumes of the medial rectus (1275 mm^3^ vs. 888 mm^3^, *p* < 0.01), lateral rectus (950 mm^3^ vs. 749 mm^3^, *p* < 0.01), superior muscle group (1868 mm^3^ vs. 1391 mm^3^, *p* < 0.01), inferior rectus (1355 mm^3^ vs. 890 mm^3^, *p* < 0.01), superior oblique (415 mm^3^ vs. 320 mm^3^, *p* = 0.02) and sum of all muscles (6368 mm^3^ vs. 4674 mm^3^, *p* < 0.01) were significantly higher in the asymmetric orbit compared to its contralateral orbit (Fig. 1). There was no significant difference in the inferior oblique (503 mm^3^ vs. 436 mm^3^, *p* = 0.15) muscle volume in the asymmetric vs. contralateral orbit. In patients without asymmetric disease, there was no significant difference in the individual muscles volumes in the right orbit compared to the left orbit.

## Discussion

Asymmetric TED is a reasonably common occurrence in the course of TED and has been identified as a significant contributor to psychosocial distress and impaired quality of life [5]. Although TED is typically bilateral and symmetric, TED should be considered in the work up of asymmetric orbital inflammatory disease.

Asymmetric TED has been reported to have a prevalence of 9–41% of patients [1, 2]. Our findings report a prevalence of 33%. The reported prevalence of asymmetric TED varies across studies due to differences in the definition of asymmetry, the clinical stage of disease and inter-observer variations in the clinical assessments. Studies that have evaluated patients later in the course of disease are likely to have a lower prevalence of asymmetric disease, as some patients with asymmetric disease go on to develop symmetric disease [2]. Nevertheless, it is important to keep asymmetric TED in the differential list of patients with asymmetric orbital disease.

The epidemiology of asymmetric TED has been studied previously, with conflicting results. Some studies suggest that asymmetric disease is more prevalent in males, whilst others have failed to show such a difference [1, 6]. Asymmetry has also been associated with older age in a previous study, although most previous studies have not commented on associations with age [6, 7]. Our study did not find a difference in the gender or sex composition of asymmetric TED patients compared to symmetric TED patients.

The pathophysiology of asymmetric TED has not been elucidated, and several potential mechanisms have been proposed including asymmetric distribution of antigen, and anatomical differences causing differential blood flow or lymphatic drainage [3]. One study investigated whether sleeping position may be associated with asymmetric disease and did not find an association [7]. Normal anatomic differences between orbits (such as differences in globe protrusion) may become more pronounced in the setting of active orbital inflammation, manifesting as asymmetric disease. Our study found the found the asymmetric orbit to have significantly higher muscles volumes compared to its contralateral orbit, thus muscle expansion is one of the likely contributing factors to the development of asymmetric proptosis.

Asymmetric disease has been associated with higher clinical activity scores, and more severe disease [6, 8]. Perros et al. [6] reported that asymmetric patients had a mean CAS of 3.0 compared to symmetric patients who had a mean CAS of 1.7. This may be due to the fact that asymmetric disease is more common in the earlier stages of TED [2]. Regardless, the treatment of asymmetric TED does not differ from symmetric TED. Other reported associations of asymmetric disease include euthyroid/hypothyroid status. Eckstein et al. [9] reported a prevalence of 23% of asymmetric disease in euthyroid/hypothyroid patients, defined by a proptosis difference of greater than or equal to 3 mm, compared to 4.8% in hyperthyroid patients. We however did not find a significant correlation between asymmetry and thyroid status, anti-TPO levels or anti-TSH receptor antibody levels, possibly due to type 2 error.

One of the main challenges in determining the clinical and radiological associations of asymmetric disease is the lack of a universal, standardised definition for asymmetric disease. Most studies have used a difference in Hertel measurements of either more than 2 mm or 3 mm to define asymmetric disease [1, 2, 5, 9]. Others have used definitions based on the presence of asymmetric clinical symptoms and signs such as differences in lid swelling or erythema, conjunctival redness or palpebral aperture difference of more than 2 mm [6, 7]. These definitions are based on clinical assessments, which can vary significantly between readers [10]. The lack of a universal definition likely accounts for many of the differences seen in terms of associations of asymmetric TED with age, gender, thyroid status and disease severity. A more objective definition of asymmetric disease, based on more objective markers such as radiologically derived proptosis or muscles volumes may help to standardise the definition of asymmetric TED and allow for comparisons between studies.

Limitations to this study include its retrospective nature. As a result, it was not possible to determine the inter-rater reliability of the Hertel exophthalmometry measurements.

Asymmetric TED is reasonably common and may be seen in one-third of TED patients. The extraocular muscles volumes are higher in the asymmetric orbit compared to its contralateral orbit, suggesting muscle volume expansion to be an underlying contributor to asymmetry. In future, use of more objective parameters to define asymmetric TED is required to enable reliable comparisons between studies.
